# Activation of ErbB2 and Downstream Signalling via Rho Kinases and ERK1/2 Contributes to Diabetes-Induced Vascular Dysfunction

**DOI:** 10.1371/journal.pone.0067813

**Published:** 2013-06-27

**Authors:** Saghir Akhtar, Mariam H. M. Yousif, Gursev S. Dhaunsi, Fatma Sarkhouh, Bindu Chandrasekhar, Sreeja Attur, Ibrahim F. Benter

**Affiliations:** 1 Department of Pharmacology and Toxicology, Faculty of Medicine, Kuwait University, Safat, Kuwait; 2 Department of Pediatrics, Faculty of Medicine, Kuwait University, Safat, Kuwait; University of Southampton, United Kingdom

## Abstract

Diabetes mellitus leads to vascular complications but the underlying signalling mechanisms are not fully understood. Here, we examined the role of ErbB2 (HER2/Neu), a transmembrane receptor tyrosine kinase of the ErbB/EGFR (epidermal growth factor receptor) family, in mediating diabetes-induced vascular dysfunction in an experimental model of type 1 diabetes. Chronic treatment of streptozotocin-induced diabetic rats (1 mg/kg/alt diem) or acute, ex-vivo (10^−6^, 10^−5^ M) administration of AG825, a specific inhibitor of ErbB2, significantly corrected the diabetes-induced hyper-reactivity of the perfused mesenteric vascular bed (MVB) to the vasoconstrictor, norephinephrine (NE) and the attenuated responsiveness to the vasodilator, carbachol. Diabetes led to enhanced phosphorylation of ErbB2 at multiple tyrosine (Y) residues (Y1221/1222, Y1248 and Y877) in the MVB that could be attenuated by chronic AG825 treatment. Diabetes- or high glucose-mediated upregulation of ErbB2 phosphorylation was coupled with activation of Rho kinases (ROCKs) and ERK1/2 in MVB and in cultured vascular smooth muscle cells (VSMC) that were attenuated upon treatment with either chronic or acute AG825 or with anti-ErbB2 siRNA. ErbB2 likley heterodimerizes with EGFR, as evidenced by increased co-association in diabetic MVB, and further supported by our finding that ERK1/2 and ROCKs are common downstream effectors since their activation could also be blocked by AG1478. Our results show for the first time that ErbB2 is an upstream effector of ROCKs and ERK1/2 in mediating diabetes-induced vascular dysfunction. Thus, potential strategies aimed at modifying actions of signal transduction pathways involving ErbB2 pathway may prove to be beneficial in treatment of diabetes-induced vascular complications.

## Introduction

The global incidence of diabetes mellitus is set to rise to over 550 million by 2030 [1]-[2]. Diabetes and/or the associated hyperglycemia leads to the development of cardiovascular complications such as altered vascular reactivity, hypertension, atherosclerosis, microangiopathy, ischemic heart disease, myocardial infarction and cardiac myopathy that collectively are three to eight-fold more likely in diabetic patients and represent a major cause of mortality [3]. However, the exact underlying mechanisms for the development of vascular complications such as altered vascular reactivity in diabetes are poorly understood and may involve multiple signaling pathways that are affected by hyperglycemia [4]–[5].

The epidermal growth factor receptor (EGFR or ErbB) family of receptor tyrosine kinases (RTKs) comprises four members: EGFR (ErbB1, HER1), ErbB2 (EGFR2, Neu, HER2), ErbB3 (EGFR3, HER3) and ErbB4 (EGFR4, HER4) and are regulators of important cellular functions such as cell growth, proliferation, differentiation, motility, invasivness and apoptosis [6]–[7]. ErbB2 receptor is a 175-kDa glycoprotein that lacks a known ligand and therefore relies on heterodimerization with other EGFR family members for signaling. Dimerization of ErbBs results in activation of multiple downstream signalling pathways such as the mitogenic Ras/Raf/extracellular-signal-regulated kinase 1/2 (ERK1/2), the p38 mitogen activated protein (MAP) kinase or the PI3-kinase/Akt survival pathways [7]–[10]. Alternatively, transactivation of ErbBs can occur via G-protein coupled receptors (GPCRs), such as angiotensin II (Ang II), thrombin, aldosterone and endothelin [11]–[15]. Depending on the specific cellular conditions, EGFR transactivation can occur via upstream kinases such as c-src [11] or involve metalloprotease and/or ADAM(a disintegrin and metalloprotease)-dependent shedding of cell-surface bound EGF-like ligands [14].

There is emerging evidence that the ErbB family of RTKs might act as central ‘hub’ or transducer of information from diverse signaling cascades which makes them important players in several diabetic complications. For example upregulation of EGFR activity is thought to be important in mediating renal pathologies, cardiac fibrosis and vascular dysfunction (for reviews see [6], [15]). Indeed, we have previously shown in a rat model of type 1 diabetes that EGFR levels are raised in the diabetic vasculature and chronic inhibition of EGFR with the selective receptor antagonist AG1478, prevented the development of diabetes-induced abnormal vascular reactivity in the mesenteric vascular bed and renal artery [16]–[18]. Gene expression profiling of the mesenteric vasculature showed that the correction in vascular dysfunction achieved by AG1478 was attained by blocking the up-regulation of ∼ 85% of the approximately 1100 genes whose expression had been altered in the diabetic mesenteric vasculature [18]. Betacellulin- a ligand for the EGFR family of receptors, when administered in mice also led to retinal vascular damage thereby further implicating EGFR signaling in vascular dysfunction [19]. Furthermore, our data in rats and that from a mouse model of type 1 diabetes [20] and in an experimental model of type 2 diabetes [21] support the assertion that enhanced EGFR might be a common mechanism mediating vascular dysfunction in both type 1 and type 2 diabetes. However, little is known about the role of other members of the ErbB family in diabetes-induced vascular complications. Here, we report on the role of ErbB2 in mediating diabetes-induced vascular dysfunction in an experimental model of type 1 diabetes.

RhoA is a member of a small monomeric GTPase family that is involved in smooth muscle contraction and the regulation of several other smooth muscle-dependent processes such as cell adhesion, motility, migration, and proliferation [22]–[23]. On activation, such as with Angiotensin II (AngII), RhoA is converted from the cytoplasmic, inactive GDP-bound form into an active GTP-bound complex that translocates to and binds the plasma membrane via geranylgeranylation to initiate intracellular signal transduction. The downstream effectors of RhoA are Rho-associated, coiled-coil–containing protein kinases (ROCKs). The best characterized of the ROCK isoforms are ROCK1 and ROCK2 that share 65% homology in amino acid sequence and 92% homology in their kinase domains. Both ROCK1 and ROCK2 are serine-threonine kinases that are able to maintain the phosphorylated state of the myosin light chain and thus the contractile tone independently of intracellular calcium levels [22]–[23]. RhoA/ROCK pathway over-activity has been shown to be involved in several pathophysiologic processes including angiogenesis, atherosclerosis, glomerulosclerosis, hypertension, myocardial hypertrophy and vascular remodeling [24]. Fasudil, an inhibitor of ROCKs approved for human use, has been shown to improve vascular function and remodeling including in diabetes [25]–[26]. The cardiovascular benefits associated with “statins” may also occur through inhibition of ROCKs [27]. However, the precise upstream and downstream effectors of ROCKs are not known. In this study, we hypothesised that ErbB2 is an upstream effector of ROCKs and ERK1/2 that its up-regulation leads to diabetes-induced vascular dysfunction in the mesenteric vascular bed.

## Materials and Methods

### Drugs and Reagents

Tyrphostin AG825 ((E)-3-[3-[2-Benzothiazolythio)methyl]-4-hydroxy-5-methoxyphenyl]-2-cyano-2-propen -amide), AG1478 (*N*-(3-Chlorophenyl)-6,7-dimethoxy-4-quinazolinanine hydrochloride), Fasudil hydrochloride (HA 1077), and PD98059, (2-(2-Amino-3-methoxyphenyl)-4H-1-benzopyran-4-one) were purchased from Tocris Biosciences, UK. Streptozotocin (STZ), (±) Norepinephrine-bitartrate and Carbachol were all purchased from Sigma Chemical Co. (St Louis, USA). The siRNA pool targeting rat ErbB2 mRNA (On-Target Smart Pool) as well as the non-targeting control siRNA pool (On-Target plus control pool), and DharmaFect II transfection reagent were all purchased from Dharmacon/Thermo Fisher Scientific, USA.

### In vivo Studies and Ethics Statement

Animal studies were conducted in accordance with the National Institutes of Health Guide ‘Principles of laboratory animal care’ (NIH publication no. 85–23, revised 1985) and approved by Kuwait University Health Sciences Animal Ethics Committee. Male Wistar rats weighing about 300 g were used in this study and divided into the following groups for chronic drug adminsitration (N = 12). Group 1: Non-diabetic (Control) animals, Group 2: STZ (55 mg/kg body weight)-treated diabetic (D) animals; Group 3: D+AG825- a selective inhibitor of ErbB2 tyrosine kinase, (1 mg/kg *alt diem ip*); Group 4: D+AG1478- a selective inhibitor of EGFR, (1 mg/kg *alt diem ip*). The doses used of these selective inhibitors were based on on our extensive previous studies of these drugs in the same model of diabetes [11], [16]–[18], [28]–[29]. Drugs were administered i.p. every other day for four weeks starting from day one of diabetes induction. In all cases, the last dose of the pharmacological agents was administered at 4pm and animals were sacrificed at 9am on the next day. Additional animals in Groups 1 and 2 were also used for acute drug treatments of the perfused isolated mesenteric bed (see below).

### Induction of Diabetes

STZ was used to induce diabetes as a single *ip* injection of 55 mg/kg body weight dissolved in citrate buffer (pH 4.5) whereas age-matched control rats received the citrate buffer vehicle only. Body weight and basal glucose levels were assessed prior to STZ injection and after 4 weeks just before sacrificing the animals. An automated blood glucose analyzer (Glucometer Elite XL) was used to assess blood glucose concentrations 48h after STZ injection and rats with a blood glucose concentration above 250 mg/dl were declared diabetic.

### Vascular Reactivity Studies in the Perfused Isolated Mesenteric Vascular Bed

The mesenteric vascular beds were isolated as described by us previously [16] and transferred into Petri dishes containing oxygenated Krebs’ solution. A polyethylene cannula was inserted into the mesenteric artery and the mesenteric vascular bed placed in a warm water-jacketed chamber at 37°C. The preparation was perfused with Krebs’ solution at 37°C, oxygenated with 95% oxygen and 5% carbon dioxide, delivered at a constant flow rate of 6 ml/min using a multichannel masterflex peristaltic pump. Changes in perfusion pressure which reflect peripheral resistance were measured via a pressure transducer connected to a Lectromed. The preparation was always allowed to equilibrate for at least 30 min. A bolus injection of 100 nmol NE was usually given at the beginning of the experiment as a test for tissue responsiveness. The vasoconstrictor response of NE was investigated in the perfused mesenteric vascular bed. Following the period of equilibration, successive, increasing doses (10 pmol to 10 micromol) of NE (were given at regular intervals to establish the vasoconstrictor responses (mmHg). Additionally, the vasodilator responses of carbachol (0.1 pmol to100 nmol) were also investigated in the perfused mesenteric vascular bed. Here, following equilibration, the perfused mesenteric bed was constricted by perfusion with Krebs’ solution containing sub-maximal doses of NE so as to attain a similar level of responsiveness (approx. EC70%) in both control and diabetic tissue prior to evaluating the dose-dependent responsiveness to carbachol. Thus, based on the dose response curves with NE, we used 1×10^−6^ M for control and 1×10^−7^ M for diabetic mesenteric tissue. After establishing a steady level of pre-contraction, successive increasing doses of carbachol were given at regular intervals. The vasodilator response is expressed as % of the pre-contraction induced by NE (10^−5^ M). For acute drug treatments, the named drug at stated concentrations (AG825 at 10^−5^ or 10^−6^M; PD 98509 or Fasudil at 10^−6^ M) was added to the perfusate 30 mins prior to the vasoactive agent as described previously [16].

### Western Blotting Studies

Western Blotting for total and/or phosphorylated forms of ErbB2, EGFR, ERK1/2, and Rho kinases (ROCK I and II) was performed essentially as described by us previously [11], [28]. Briefly, rat mesenteric vascular beds were isolated, snap frozen in liquid nitrogen and stored at –80°C. After defrosting on ice, tissues were lysed in buffer (pH 7.6) containing 50mM Tris-base, 5 mM EGTA, 150 mM NaCl, 1% Triton 100, 2 mM Na_3_VO_4_, 50 mM NAF, 1 mM PMSF, 20 µM phenylarsine, 10 mM sodium molybdate, 10 µg/ml leupeptin and 8 µg/ml aprotinin, centriguged and protein concentration of the collected supernatants estimated by BioRad BCA protein assay. Aliquots containing equal amounts of protein were subjected to SDS-polyacrylamide gel electrophoresis (SDS-PAGE) and transferred onto nitrocellulose membrane (Schleicher & Schuell, Dassel, Germany). Membranes were then incubated with either monoclonal antibodies (Cell Signaling, USA) to detect phosphorylated and total forms of ErbB2, EGFR (bands seen at approximately 175 kDa), ERK1/2 (at 42/44 kDa) or Rho kinases (ROCK I and II) (at 160 kDa) and subsequently with appropriate secondary antibodies conjugated to horseradish peroxidase (Amersham, UK). Immunreactive bands were detected with SuperSignal chemiluminescent substrate (Pierce, UK) using Kodak autoradiography film (G.R.I., Rayne, U.K.). To ensure equal loading of proteins β-actin levels were detected using primary rabbit anti-human β-actin antibody followed by the secondary anti-rabbit IgG horse-radish peroxidase conjugated antibody (Cell Signaling, USA). Images were finally analysed and quantified by densitometry and all data normalized to β-actin levels [11], [28]. The following antibodies from Cell Signaling (USA) were used in this study: t-Her2/ErbB2-Antibody (29D8) (rabbit) Cat. No. 2165, p-Her2/ErbB2-Antibody (Tyr877) (rabbit) Cat. No. 2241, p-Her2/ErbB2-Antibody (Tyr1248; labeled in figures as Y1248a) (rabbit) Cat. No. 2247, p-Her2/ErbB2-Antibody (Tyr1248/EGFR Tyr1173; labelled as Y1248b) (rabbit) Cat. No. 2244, p-Her2/ErbB2-Antibody (Tyr1221/1222) (rabbit) Cat. No. 2243, p-ERK1/2 (p44/42 MAP Kinase, Thr202/Tyr204) Antibody (rabbit) Cat. No. 9101, : t-EGFR-Antibody (rabbit) Cat. No. 2232, p-EGFR-Antibody (Tyr1068) (rabbit) Cat. No. 2234, and p-EGFR-Antibody (Tyr1086) (rabbit). In addition the anti-Actin rabbit polyclonal IgG (1 µl/10 ml) Cat. No. A-2066 was obtained from Sigma Chemical Co, USA. Anti-ROCK-2-Antibody (pY256) (rabbit) Cat. No. 600-401-998 from Rockland, USA; ROCK 1-Antibody (rabbit) Cat. No. 4035 from Cell Signalling,USA and ROCK 2-Antibody (rabbit) Cat. No. ab66320 from Abcam,USA.

### Receptor Co-association/Immunoprecipitation Studies

To assess ErbB receptor co-association (indicative of receptor dimerization), immunoprecipitations were performed as described by us previously [28]. Briefly, anti- human ErbB2 antibody (Cat. No. 2165; Cell Signaling, USA ) or anti-human EGFR (Cat. No. 2232; Cell Signaling, USA) or was added to 1 mg of tissue lysate sample at a dilution of 1∶50, and incubated on a tube rotator overnight at 4°C. Then 50 µl of protein A-agarose beads (Millipore, USA) was added to the samples and incubated on an end-over-end tube rotator at 4°C for a further 3 h. The bead pellets were then washed 3 times with excess lysis buffer containing protease inhibitor cocktail (Sigma,USA) (400 µl), and collected by centrifugation at the maximum speed for 5 seconds. The pellets were then resuspended in 30 µl of sample-loading buffer and and heated to 100°C for 10 min. Samples were subjected to SDS-PAGE electrophoresis and immnunoblotting was performed for EGFR or ErbB2 as described [28].

### Vascular Smooth Muscle Cell Culture Studies

Thoracic aortas taken from untreated, non-diabetic control male Wistar rats were subjecteed to enzymatic dissociation to obtain primary rat aortic smooth muscle cell (VSMC) cultures were obtained by of the as described by us previously [11]. The obtained VSMC were utilized between *passages 3* and *10*. For the signaling studies, VSMC were initially cultured in 10% serum containing DMEM media until 60–70% confluence in T-25 flasks and then in serum-free DMEM media containing either normal (5.5 mM) or high (25 mM) D-glucose and/or co-treated with different doses of the named drugs or siRNAs for 72 h. The siRNAs targeting rat ErbB2 mRNA (On-Target Smart Pool) or the non-targeting control siRNAs (On-Target plus control pool) at either 25 nM or 50 nM were transfected using DharmaFect II transfection reagent as per the manufacturer’s instructions (Dharmacon/Thermo Fisher Scientific, USA). Cells were then lysed and equivalent amounts of proteins subjected to SDS-PAGE and immunoblotting as described for the mesenteric bed above.

### Statistical Analysis

Results were analyzed using Graph-pad Prism software. Data are presented as Mean± S.D. of ‘N’ number of experiments. Mean values were compared using analysis of variance followed by post hoc test (Bonferroni). The difference was considered to be significant when p value was less than 0.05.

## Results

### Hyperglycemia and Animals’ Body Weights

Induction of diabetes by a single i.p injection of STZ resulted in hyperglycemia that persisted in the diabetic animals and was 590±13 mg/dl at four weeks compared with 98±4 mg/dl in control animals. There was a significant reduction in the weights of diabetic rats compared to non-diabetic control animals (158±4g and 240±13 g; respectively, p<0.05). Chronic AG825 treatment *alt diem* for 4 weeks commencing from the onset of diabetes did not reverse the weight loss (165±9g) nor significantly alter blood glucose levels (583±11 mg/dl) in diabetic rats.

### Chronic or Acute Treatment with AG825 Attenuates Diabetes-induced Vascular Dysfunction in the Mesentric Vascular Bed

**Figure 1:**
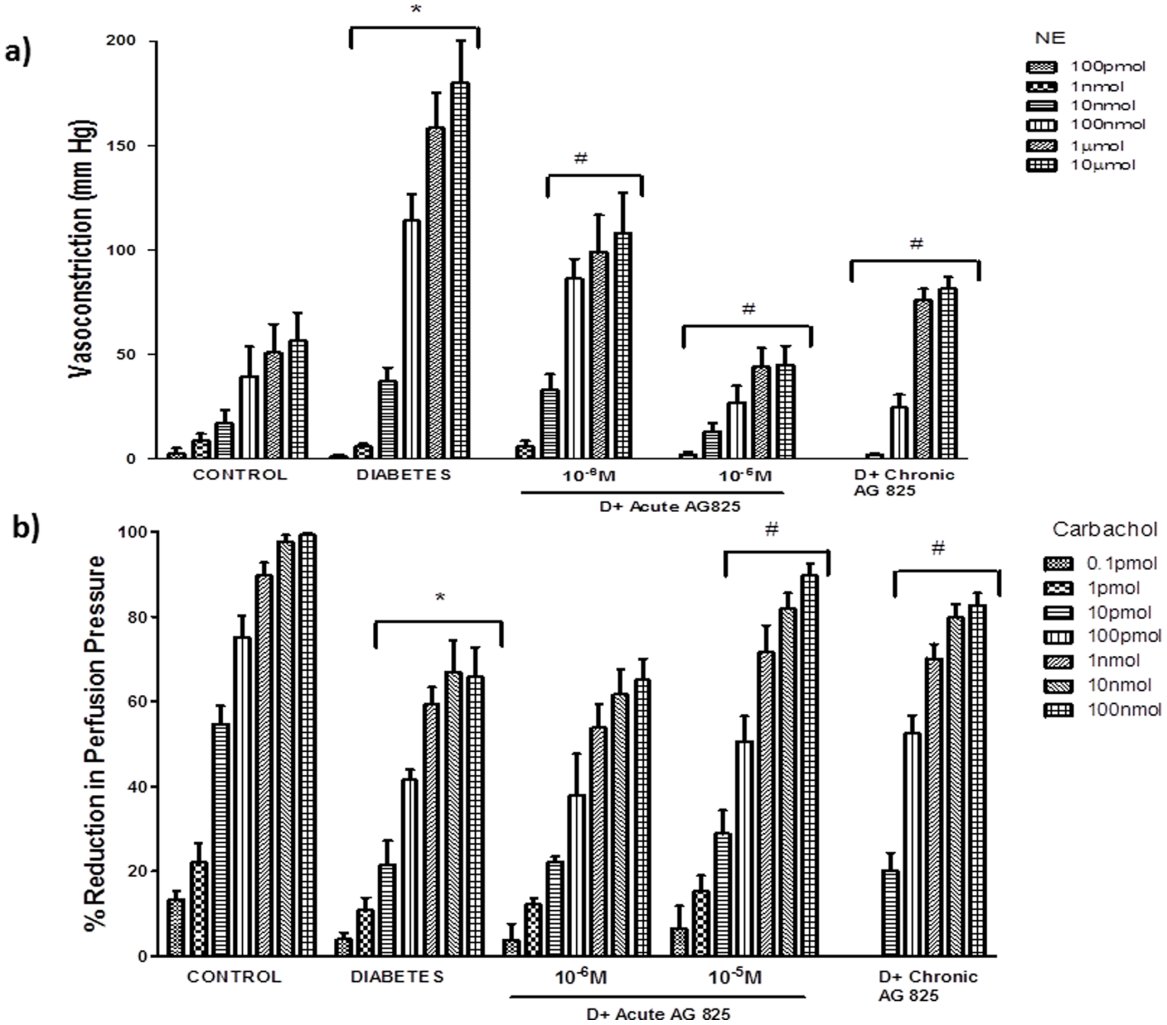
ErbB2 inhibition corrects diabetes-induced vascular dysfunction in mesenetric vascular bed. The responsiveness of the isolated perfused mesenteric vascular bed from non-diabetic control and diabetic animals to stated doses of **a)** Norephinephrine (NE) or **b)** Carbachol is shown following acute AG825 treatment (10^−6^, 10^−5^ M; D+Acute AG825) or in mesenteric vascular beds isolated from diabetic animals receiving chronic, 4-week administration of AG825 (1 mg/kg/*alt-diem*; D+Chronic AG825). Mean±S.D; N = 6; Mean values were compared using analysis of variance followed by post hoc test (Bonferroni). Asterisk (*) indicates significantly different (p<0.05) mean values from normal non-diabetic rats (Control) whereas hash (#) indicates significantly different mean values (p<0.05) from diabetic rats (Diabetes; D).

Perfused mesenteric vascular beds isolated from diabetic compared to non-diabetic control rats exhibited significantly enhanced responsiveness to the vasoconstrictor, NE (10 pmol to 10 micromol), and attenuated responsiveness to the vasodilator, carbachol (0.1 pmol to100 nmol), indicative of vascular dysfunction (p>0.05; Figure 1a and 1b). Chronic treatment of streptozotocin-induced diabetic rats (1 mg/kg/*alt diem*) or acute, ex-vivo (10^−6^, 10^−5 ^M) administration of AG825 to isolated perfused mesenteric beds from diabetic rats significantly reversed the diabetes-induced changes in responsiveness to NE (Figure 1a) and to carbachol (p>0.05; Figure 1b).

### AG825 Treatment Attenuates Diabetes-induced Elevation in ErbB2, ERK1/2 and ROCK Signaling in the Mesenteric Bed

**Figure 2:**
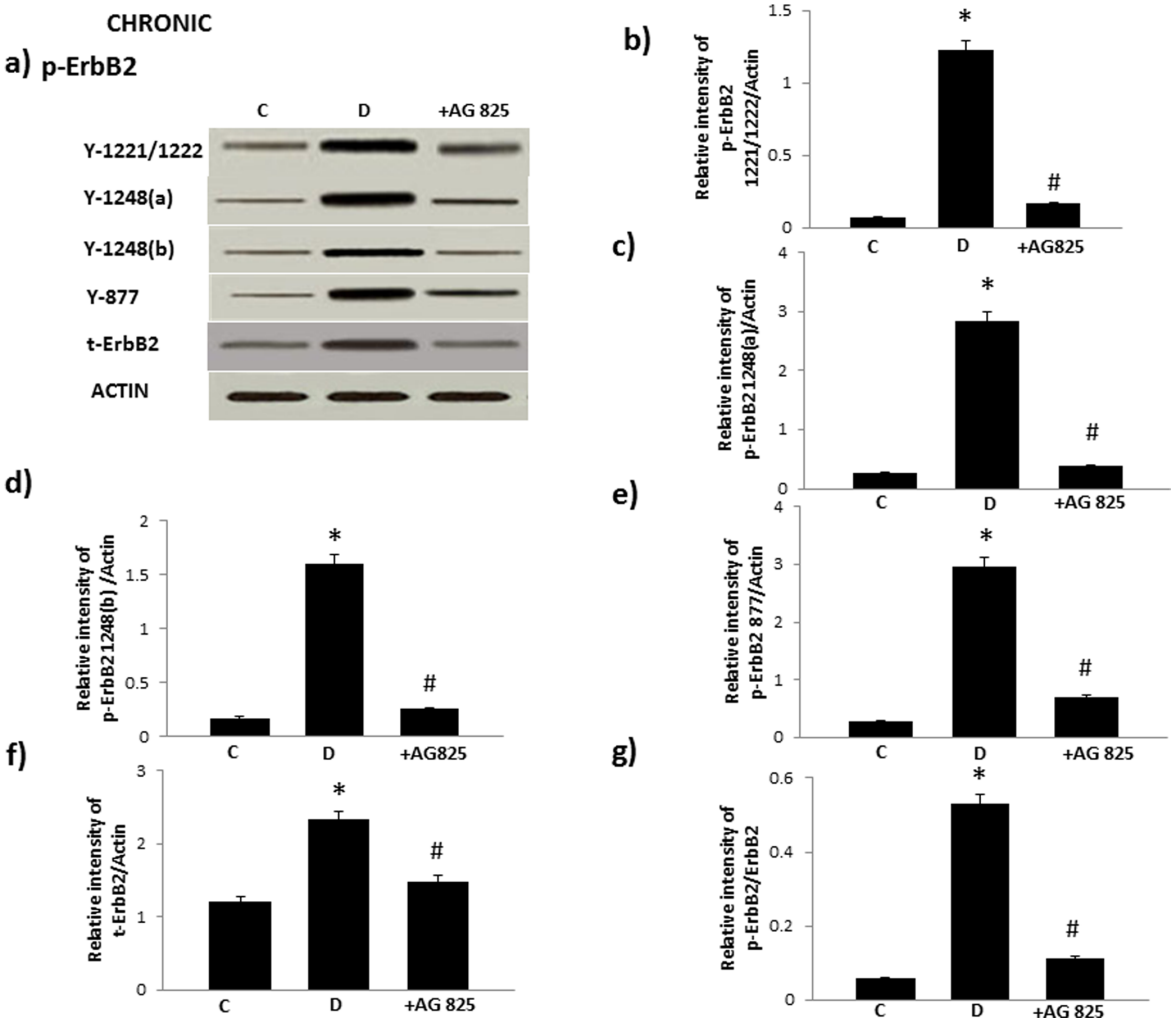
Diabetes–induced phosphorylation of ErbB2 in the mesenteric bed vasculature occurs at multiple tyrosine residues that can be attenuated by chronic treatment AG825. Panel a) is a representative Western blot showing the levels of phosphorylated ErbB2 (p-ErbB2) at the indicated tyrosines Y1221/1222, Y1248 (detected by separate antibodies labeled as Y1248 a and Y1248b, Y877, total (t-) ErbB2 and β-actin in the isolated mesenteric bed from normal controls (C), diabetic (D) and diabetic animals treated for 4 weeks with AG825 (1 mg/kg/*alt-diem*; +AG825). Panels b-e) are densitometry histograms showing levels of phosphorylated EGFR at the stated tyrosine residue and panel f) t-ErbB2 normalized to actin whereas panel g) shows the ratio of p-ErbB2 (Y1221/1222) to t-ErbB2. N = 5; Mean±SD. Asterisk (*) indicates significantly different (p<0.05) mean values from normal non-diabetic rats (C) whereas hash (#) indicates significantly different mean values (p<0.05) from diabetic rats (D).

**Figure 3:**
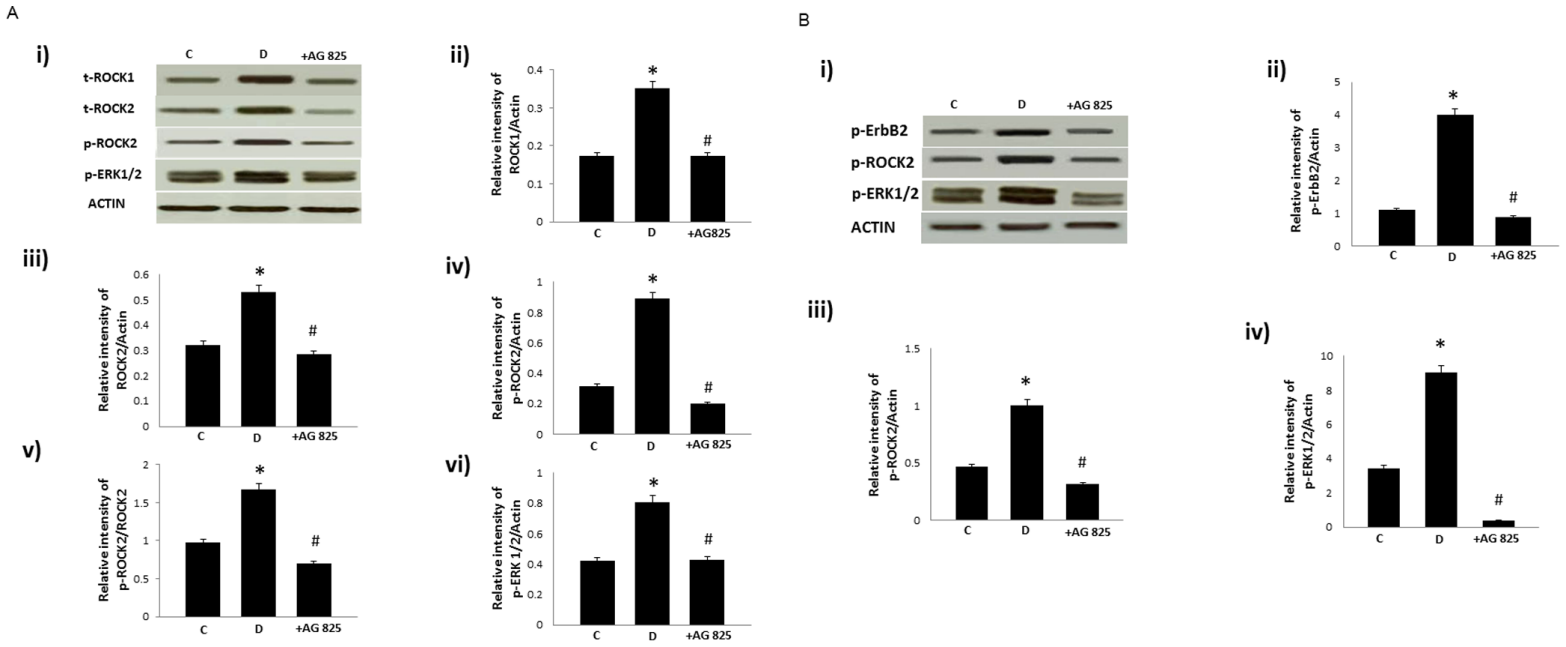
The effect of chronic (A) or acute (B) treatment with AG825 on ROCKs and ERK1/2 signaling in the isolated mesenteric bed of diabetic rats. A) Panel i) is a representative Western blot showing total (t−) or phosphorylated (p−) levels of the stated proteins in the isolated mesenteric bed from normal controls (C), diabetic (D) and diabetic animals treated either chronically (1 mg/kg/*alt-diem*) for 4 weeks (shown in figure 3A) or acutely (10^−5^M) (shown in 3B) with AG825 (+AG825); Panels ii-vi are densitometry histograms showing total (t−) or phosphorylated (p−) levels of the stated proteins normalized to actin. N = 5; Mean±SD. Asterisk (*) indicates significantly different (p<0.05) mean values from normal non-diabetic rats (C) whereas hash (#) indicates significantly different mean values (p<0.05) from diabetic rats (D).

Diabetes led to enhanced phosphorylation of ErbB2 at multiple tyrosine (Y) residues (Y1221/1222, Y1248, as detected by two different antibodies indicated as Y1248a and Y1248b, and Y877) that could be attenuated by AG825 treatment (Figure 2). Furthermore, diabetes resulted in elevated levels of total ROCK I and ROCK II as well as phosphorylated levels of ROCK II at Y256 (note at the time of peforming this study no phospho-specific antibody for detecting ROCK 1 was available) and ERK1/2 in the mesenteric vascular bed that could be significantly attenuated by chronic AG825 treatment (Figure 3a). Acute AG825 administration (10^−5 ^M) also opposed the diabetes-induced elevation in phosphorlated ErbB2 (Y1221/1222), ROCK II (Y256) and ERK1/2 in the isolated mesenteric vascular bed (Figure 3b).

### ErbB2 Co-associates with EGFR in the Diabetic Mesenteric Vascular Bed

**Figure 4:**
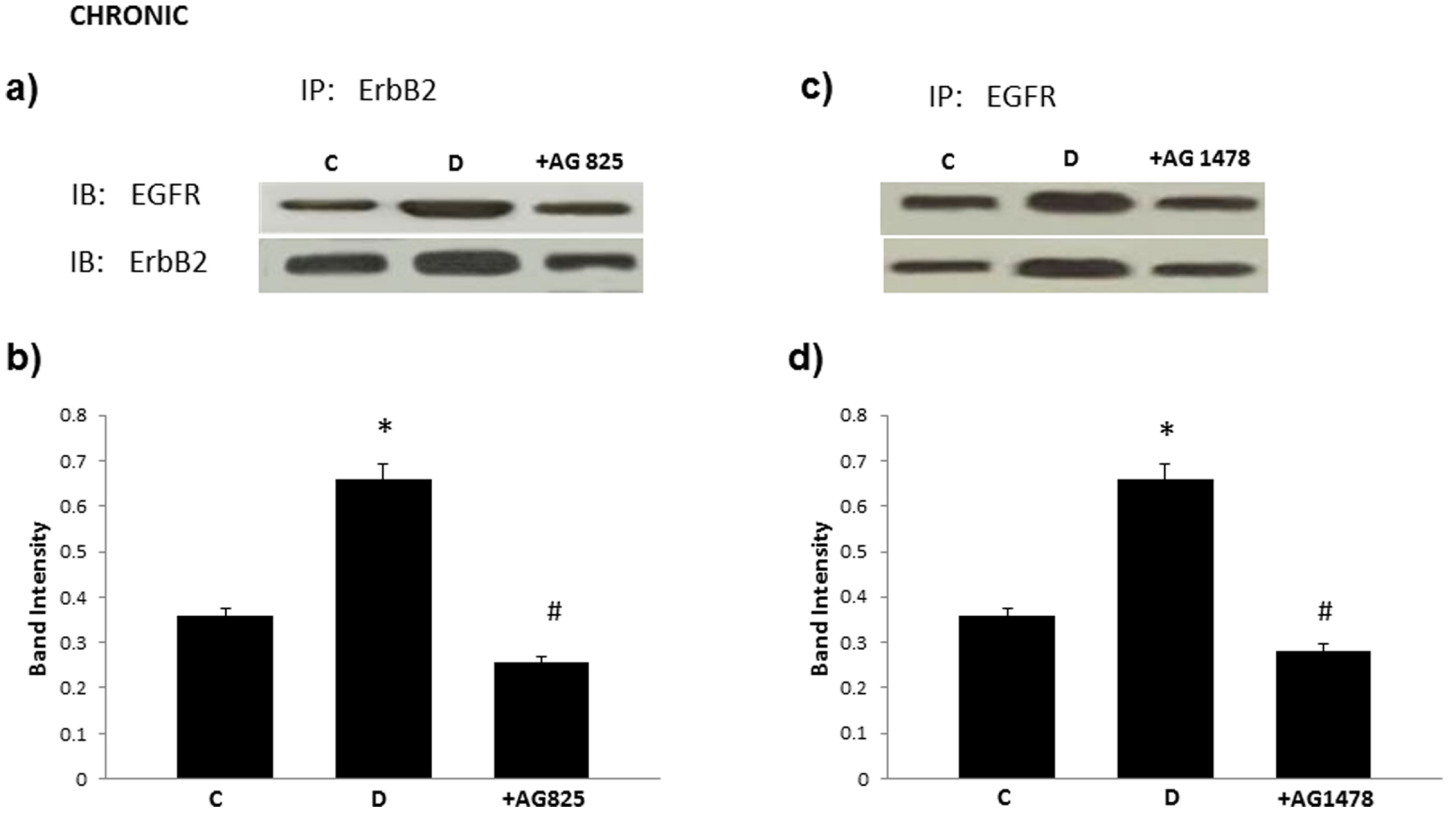
ErbB2 co-associates and forms heterodimers with EGFR in the diabetic mesenteric vascular bed that can be prevented by chronic treatment with AG825 or AG1478. Panel a) and c) are represenatative Western Blots following immunoprecipitations (IP) with either total-erbB2 or total-EGFR antibody and subsequent immunoblotting (IB) with both antibodies individually. Panel b) and d) represents the mean ratio of erbB2/EGFR dimers as assessed by densitometry for non-diabetic controls, (C), diabetic (D) and diabetic animals chronically treated with AG825 (+AG825) or AG1478 (+ AG1478) (both at dose of 1 mg/kg/*alt-diem*). N = 4; Asterisk (*) indicates significantly different (p<0.05) mean values from normal non-diabetic rats (C) whereas hash (#) indicates significantly different mean values (p<0.05) from diabetic rats (D).

Immunopreciptation of ErbB2 with an ErbB2-specific antibody resulted in co-preciptation of EGFR and similarly, pull down of EGFR with a EGFR-specific antibody showed co-association with ErbB2 (Figure 4). The level of co-association of these two ErbB receptors was elevated in diabetes and blocked by chronic AG825 or AG1478 administration (both at dose of 1 mg/kg/*alt-diem*) in diabetic animals (Figure 4).

### Acute or Chronic Treatment with AG1478, a Selective Inhibitor of EGFR, also Attenuates Diabetes-induced Elevation in ROCK and ERK1/2 Signaling in the Mesenteric Vascular Bed

**Figure 5:**
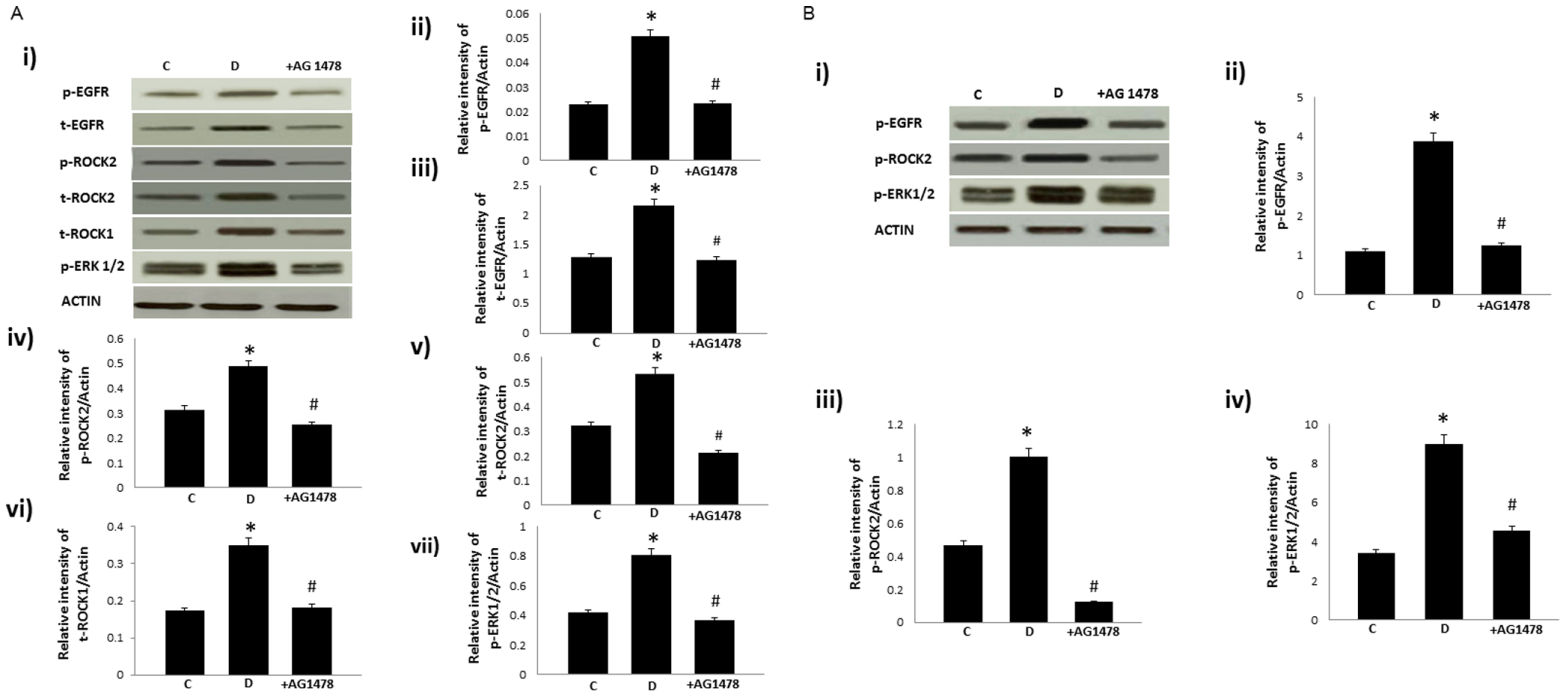
Chronic (A) or acute (B) treatment with AG1478, a selective inhibitor of EGFR, attenuates diabetes-induced elevation in ROCK and ERK1/2 signaling in the mesenteric vascular bed. A) Panel i) is a representative western blot showing total (t−) or phosphorylated (p−) levels of the stated proteins in the isolated mesenteric bed from normal controls (C), diabetic (D) and diabetic animals treated for 4 weeks with AG1478 (1 mg/kg/*alt-diem*; +AG825); Panels ii–vii) are densitometry histograms showing total (t-) or phosphorylated (p−) levels of the stated proteins normalized to actin. B) Panel i) is a representative western blot showing phosphorylated (p−) levels of the stated proteins in the isolated mesenteric bed from normal controls (C), diabetic (D) and diabetic animals treated for 4 weeks with AG1478 (1 mg/kg/*alt-diem*; +AG825); Panels ii–iv) are densitometry histograms showing phosphorylated (p−) levels of the stated proteins normalized to actin. N = 5; Mean±SD. Asterisk (*) indicates significantly different (p<0.05) mean values from normal non-diabetic rats (C) whereas hash (#) indicates significantly different mean values (p<0.05) from diabetic rats (D).

At doses that have previously been shown to prevent diabetes-induced vascular dysfunction (Benter et al, 2005a), AG1478 administered chronically (Figure 5a) or acutely (Figure 5b), also attenuted the diabetes-induced changes in total and/or phosphorylated EGFR, ROCK and ERK1/2.

### Acute Treatments with Fasudil or PD98059 Inhibit ROCK and ERK1/2 Signaling Respectively and Attenute Diabetes-induced Vascular Dysfunction in the Mesenteric Vascular Bed

**Figure 6:**
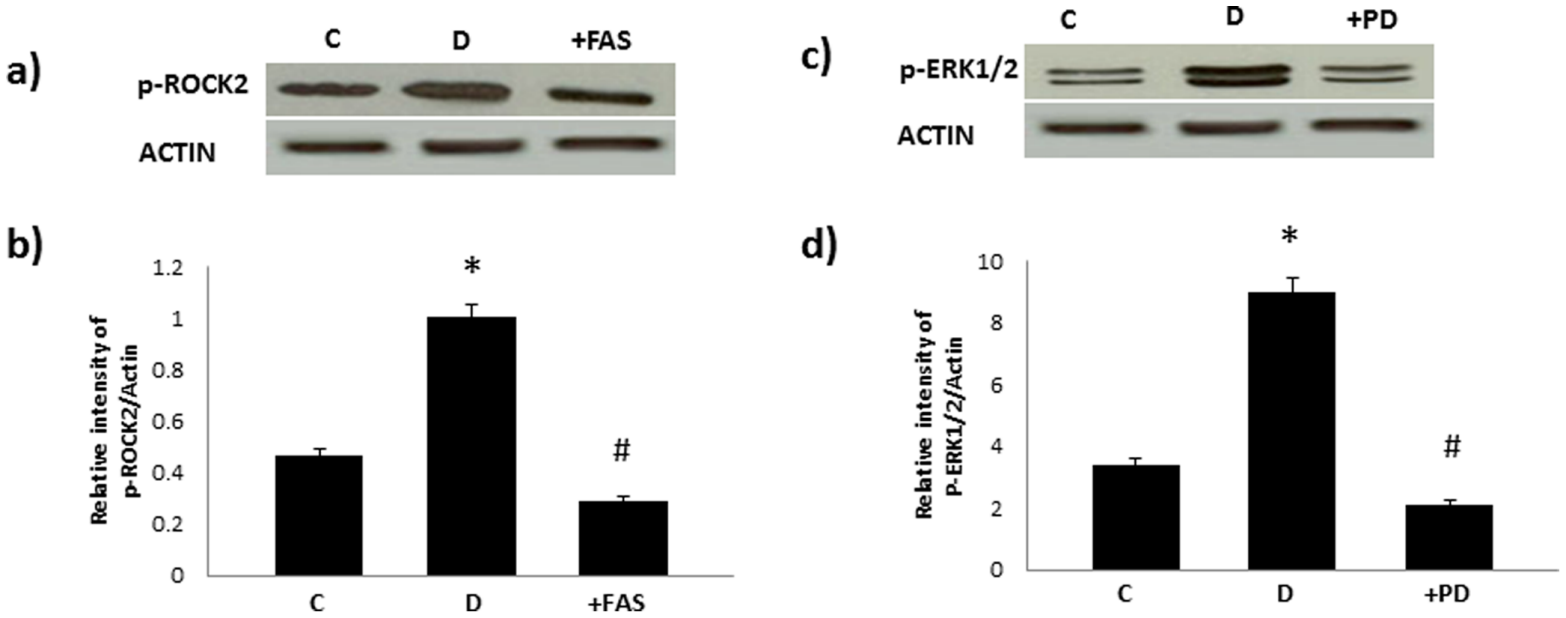
Acute treatment with Fasudil (panels a and d) or PD98059 (b and c) attenuates diabetes-induced elevation in phosphorylation of ROCK and ERK1/2 in the mesenteric vascular bed. Panels a) and c) are representative Western blots showing phosphorylated (p-) levels of the stated proteins in the isolated mesenteric bed from normal controls (C), diabetic (D) and diabetic mesenteric bed treated with fasudil (10^−6^M;+FAS) or PD 98509 (10^−6^M;+PD) respectively. Panels b) and d) are densitometry histograms showing phosphorylated (p-) levels of the stated proteins normalized to actin. N = 5; Mean±SD. Asterisk (*) indicates significantly different (p<0.05) mean values from normal non-diabetic rats (C) whereas hash (#) indicates significantly different mean values (p<0.05) from diabetic rats (D).

**Figure 7:**
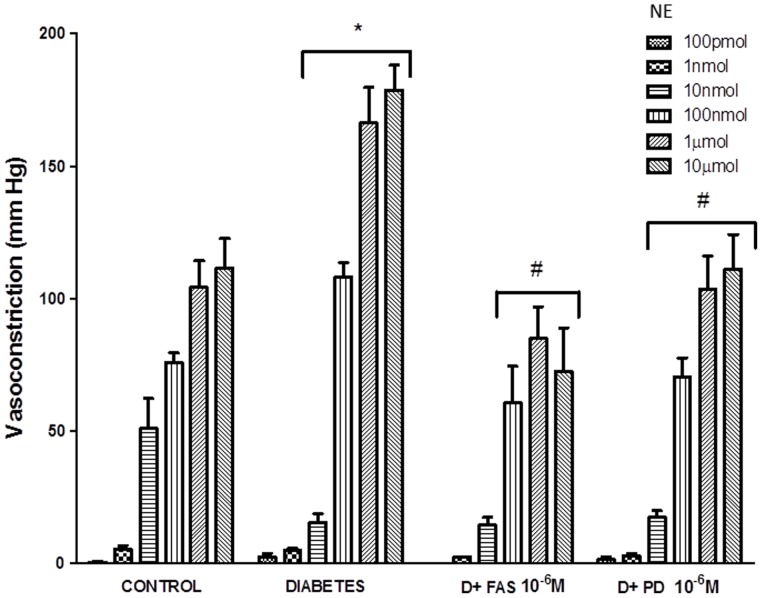
Acute treatments with Fasudil or PD98059 attenuate the enhanced responsiveness of the diabetic mesenteric vascular bed to norephinephrine. The responsiveness of the isolated perfused mesenteric vascular bed from non-diabetic control and diabetic animals to stated doses of Norephinephrine (NE) are shown following acute treatment with Fasudil (10^−6 ^M; D+FAS) or PD98059 (10^−6^ M; D+PD). Mean+S.D; N = 6; Mean values were compared using analysis of variance followed by post hoc test (Bonferroni). (* Significantly different from control, # significantly different form diabetes).

Figure 6 confirmed that acute adminsitration of Fasudil (an inhibitor of ROCKs) and PD98059 (a selective ERK1/2 inhibitor) inhibited phosphorylation of ROCK II and ERK1/2 respectively in the diabetic mesenteric vascular bed. Furthermore, the enhanced responsiveness of the perfused diabetic mesenteric vascular bed to NE was significantly attenuated by acute treatment with Fasudil or by PD98059 (Figure 7).

### High Glucose-induces Enhanced Phosphorylation of ErbB2, ROCK and ERK1/2 Signaling that can be Attenuated by AG825 or Anti-ErbB2 siRNA in VSMC

**Figure 8:**
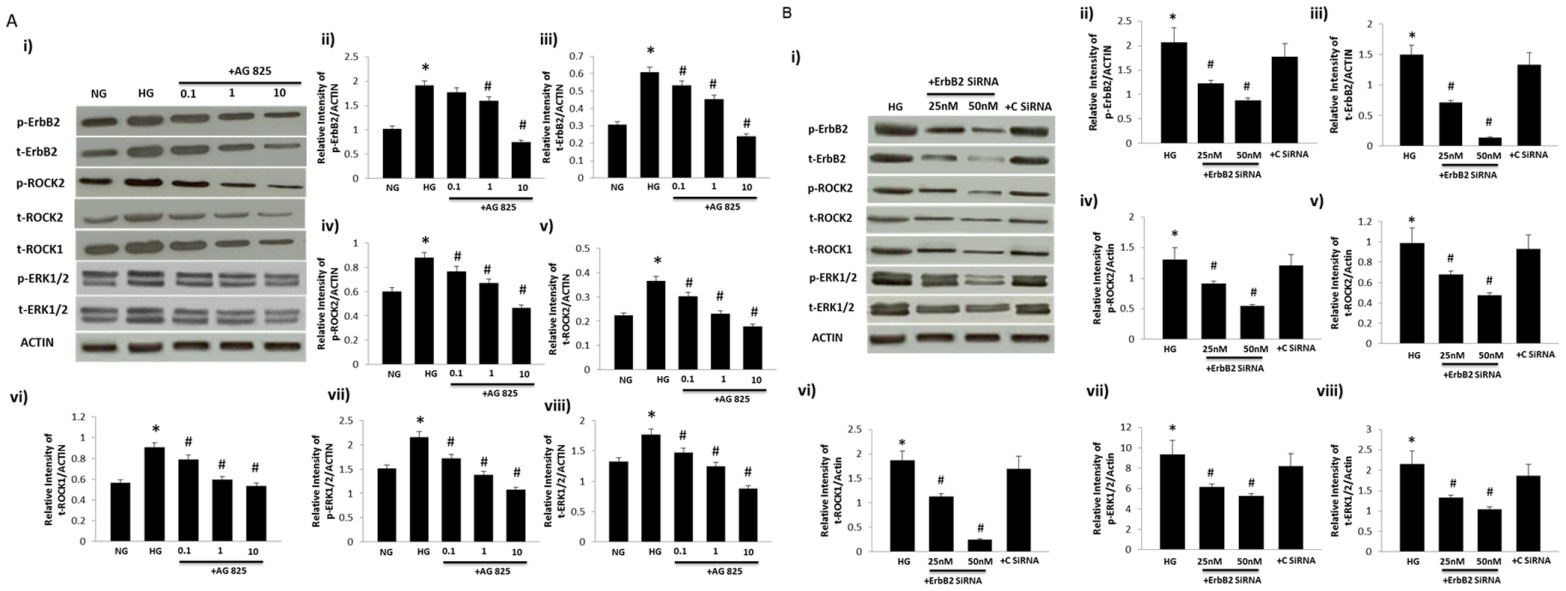
High glucose-induces phosphorylation of ErbB2, ROCK and ERK1/2 signaling that can be attenuated by treatment with (A) AG825 or (b) anti-ErbB2 siRNA in VSMC. **A) Panel i) is a representative Western blot showing total (t-) or phosphorylated (p) levels of the stated proteins in VSMC grown in normal (5.5mM) D-glucose (NG), high glucose (25.5mM D-glucose; HG) or HG cotreated with increasing micromolar doses of AG825 (+ AG825).** Panels ii-viii) are densitometry histograms showing total (t-) or phosphorylated (p-) levels of the stated proteins normalized to actin. **B)** Panel i) is a representative Western blot showing total (t-) or phosphorylated (p) levels of the stated proteins in VSMC grown in high glucose (25.5mM D-glucose; HG) or HG cotreated with increasing doses of anti-ErbB2 siRNA (ErbB2 siRNA) or non-targeting control siRNA (C siRNA). Panels ii-viii) are densitometry histograms showing total (t-) or phosphorylated (p-) levels of the stated proteins normalized to actin. N = 5; Mean±SD. Asterisk (*) indicates significantly different (p<0.05) mean values from normal non-diabetic rats (C) whereas hash (#) indicates significantly different mean values (p<0.05) from diabetic rats (D).

Cultured VSMC grown in high glucose (25 mM) for 72h led to elevated levels of total and/or phosphorylated ErbB2, ERK1/2, ROCK1 and ROCKII that were dose dependently blocked by treatment with AG825 (Figure 8a) or by anti-ErbB2 siRNA but not by non-targeting control siRNA (Figure 8b).

### High Glucose-induced ROCK and ERK1/2 Signaling can also be Blocked by an Inhibitor of EGFR in VSMC

**Figure 9:**
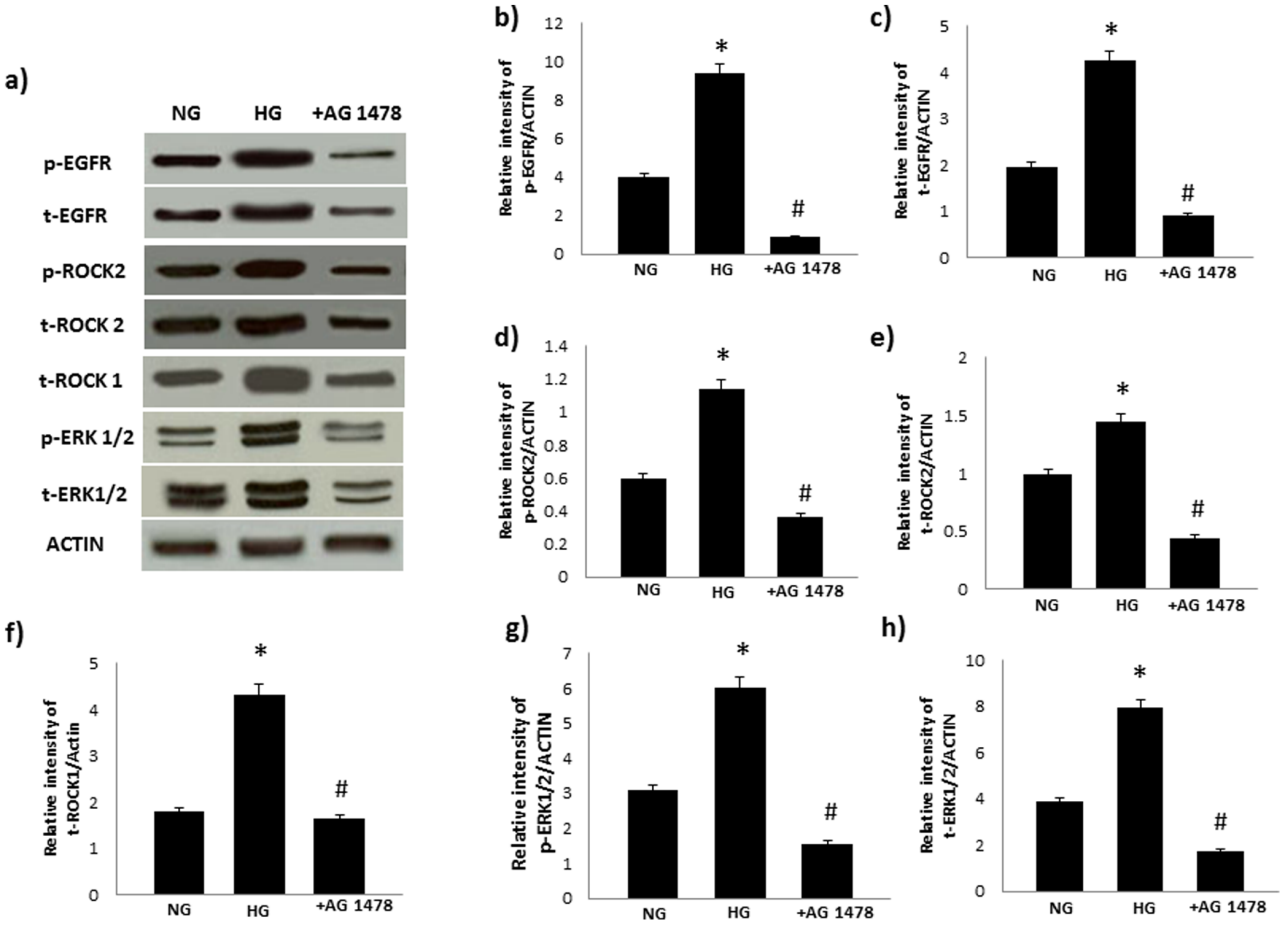
High glucose-induced changes in ROCKs and ERK1/2 can be blocked by AG1478, a selective inhibitor of EGFR, in VSMC. **Panel a) is a representative Western blot showing total (t-) or phosphorylated (p) levels of the stated proteins in VSMC grown in normal (5.5mM) D-glucose (NG), high glucose (25.5mM D-glucose; HG) or HG cotreated with AG1478 (+ AG1478).** Panels **b-h)** are densitometry histograms showing total (t-) or phosphorylated (p-) levels of the stated proteins normalized to actin. N = 5; Mean±SD. Asterisk (*) indicates significantly different (p<0.05) mean values from normal non-diabetic rats (C) whereas hash (#) indicates significantly different mean values (p<0.05) from diabetic rats (D).

Incubation of VSMC in high glucose (25mM) led to increased levels of total and/or phosphorylated EGFR, ERK1/2, ROCK1 and ROCKII that were blocked by treatment with AG178 in VSMC (Figure 9).

### ROCK is a Downstream Stream Effector of ErbB2/ERK1/2 Signaling in Diabetes-induced Vascular Dysfunction

**Figure 10:**
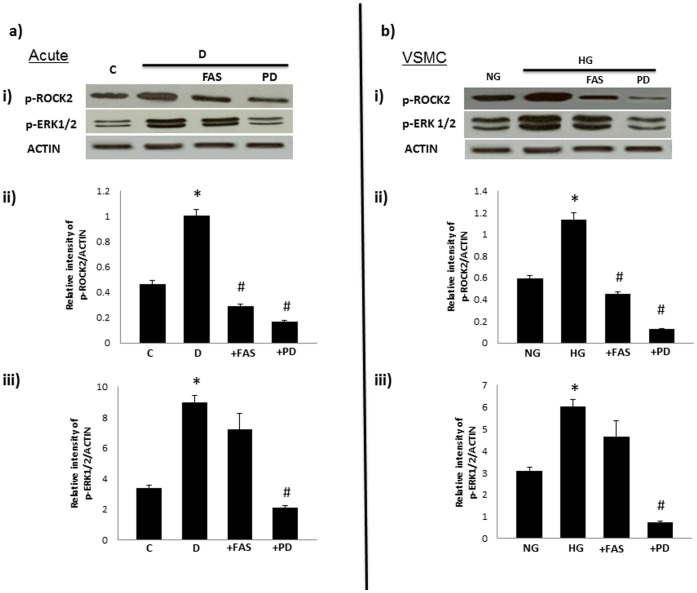
ROCK is an downstream stream effector of ERK1/2 signaling in diabetes-induced vascular dysfunction. The effect of treatment with fasudil (FAS) or PD98509 (PD) acutely in the isolated mesenteric vascular bed (A) or B) in cultured VSMC on phosphorylation (p-) of ROCKII and ERK1/2. Shown in panel i) of each figure is a respresentative Western blot and in panels ii-iii) are the densitometry histograms showing phosphorylated (p-) levels of the stated proteins normalized to actin. C = non-diabetic controls, D = Diabetic rats; NG and HG refers to VSMC grown in normal (5.5mM) D-glucose or high glucose (25.5mM D-glucose) respectively. N = 5; Mean±SD. Asterisk (*) indicates significantly different (p<0.05) mean values from normal non-diabetic rats (C) whereas hash (#) indicates significantly different mean values (p<0.05) from diabetic rats (D).

PD98059 treatment significantly blocked ERK1/2 and ROCKII phosphorylation whereas Fasudil treatment significantly attenuated diabetes-induced elevation in ROCKII but not ERK1/2 phosphorylation following acute treatment of diabetic mesenteric vascular bed (Figure 10a) or in cultured VSMC grown in high glucose (Figure 10b).

## Discussion

The underlying mechanisms leading to diabetes induced-vascular dysfunction are not well characterized. This study has identified a novel signaling pathway involving activation of ErbB2 receptor tyrosine kinase and downstream signaling via ERK1/2 and ROCK as an important mediator of diabetes-induced vascular dysfunction in an experimental model of type 1 diabetes. Thus, our data suggest that strategies targeting ErbB2 may represent novel approaches for the treatment of vascular complications associated with diabetes.

After 4 weeks of STZ-induced diabetes, vascular dysfunction was evidenced by altered responsiveness of the perfused mesenteric vascular bed mesenteric to NE and Carbachol (Figure 1) and these data are consistent with our previous reports [16]–[17]. The fact that acute or chronic treatment with AG825, a specific inhibitor of ErbB2 phosphorylation, led to normalization of the altered vasoconstrictor and vasodilator responses in the diabetic mesenteric vascular bed (Figure 1) implied that ErbB2 signaling was an important contributing pathway in the development of vascular dysfunction associated with diabetes. However, the fact that chronic administration of AG825 did not significantly correct hyperglycemia in diabetic rats implied that this pathway is not important in regulating blood glucose levels. Western blot analyses showed for the first time that diabetes led to enhanced ErbB2 expression and phosphorylation at multiple tyrosine residues (Y1221/1222, Y1248 and Y877) in the mesenteric vascular bed that was effectively blocked by chronic AG825 treatment of animals (Figure 2) confirming the importance of ErbB2 activation in diabetes-induced vascular dysfunction.

We next hypothesized that ErbB2 might be an upstream effector of ERK1/2 and ROCKs in mediating vascular dysfunction. Both ERK1/2, known downstream effectors of ErbB family of receptors, and ROCKs have been implicated in diabetes-induced vascular complications [11], . The fact that diabetes-induced elevation in ROCK expression and phosphorylation at Y256 as well as ERK1/2 phosphorylation were markedly attenuated by chronic treatment with AG825 (Figure 3A) supported our assertion that ErbB2 is an upstream effector of ERK1/2 and ROCKs in mediating vascular dysfunction in diabetes. The fact that acute treatment of the diabetic mesenteric bed vasculature with AG825 also led to effective inhibition of diabetes-induced elevation in ErbB2, ROCK and ERK1/2 phosphorylation (Figure 3B) as well as correction of the altered vascular reactivity to NE and carbachol (Figure 1) not only supported our hypothesis further but also implied that ErbB2 blockade might be effective in both a preventative (chronic administration) or short-term (acute) treatment strategy in diabetes-induced vascular dysfunction.

We have previously shown that EGFR, another member of the ErbB family of RTKs, is also involved in mediating diabetes-induced vascular dysfunction [11], [16]–. Furthermore, given the fact the ligand-less ErbB2 typically requires another ErbB receptor as a dimerization partner for effective signaling, and that in cancer biology, ErbB2 is known to be the preferred heterodimerization partner for EGFR [10], [30], we hypothesized that activation of ErbB2 might also involve heterodimerization with EGFR. Evidence in support of our hypothesis was provided by immunoprecipitation studies where co-association of the two receptors was observed in the diabetic mesenteric vascular bed (Figure 4). Further, elevated levels of receptor co-association associated with diabetes could be blocked by chronic treatment with either AG825 or AG1478, a selective inhibitor of EGFR tyrosine kinase activity, at dosing regimens that prevented diabetes-induced vascular dysfunction (Figure 1 and refs [16]–[17]). Although our data does not rule out the possibility of ErbB2 heterodimerization with other family members, the fact that both diabetes-induced elevation in ROCK and ERK1/2 activity in the mesenteric vascular bed was also markedly corrected by chronic or acute EGFR inhibition (Figures 5) further supported our notion that ErbB2/EGFR heterodimerization with subsequent activation of ROCK and ERK1/2 led to diabetes-induced vascular dysfunction.

In cultured VSMC, high glucose-induced elevations in phosphorylated ErbB2, ROCK (Y256) and ERK1/2 were dose-dependently reversed by AG825 treatment (Figure 8A) as well as by siRNA targeting ErbB2 mRNA (Figure 8B). These in vitro results support out findings in vivo and further imply that hyperglycemia associated with diabetes leads to elevation in ErbB2 expression and activity that ultimately mediates vascular dysfunction. The fact that EGFR blockade by AG1478 treatment also prevented high glucose-induced elevation in ROCK and ERK1/2 phosphorylation in VSMC (Figure 9) provided further supporting evidence that both ErbB2/EGFR heterodimerization is likely to be upstream of ROCK and ERK1/2 signaling.

To confirm the role of ROCK and ERK1/2 as mediators of diabetes-induced vascular dysfunction, we next hypothesized that blockade of ROCK signaling via fasudil, or ERK1/2 signaling by PD 98059 should correct the hyper-reactivity of the diabetic mesenteric vascular bed to NE (Figures 6 and 7). Both agents corrected the enhanced NE responsiveness of the mesenteric bed isolated from diabetic rats in a manner similar to that observed for AG825 (Figure 1a) confirming their importance in diabetes-induced vascular dysfunction consistent with recent reports from other groups [26], [31]–[32].

**Figure 11:**
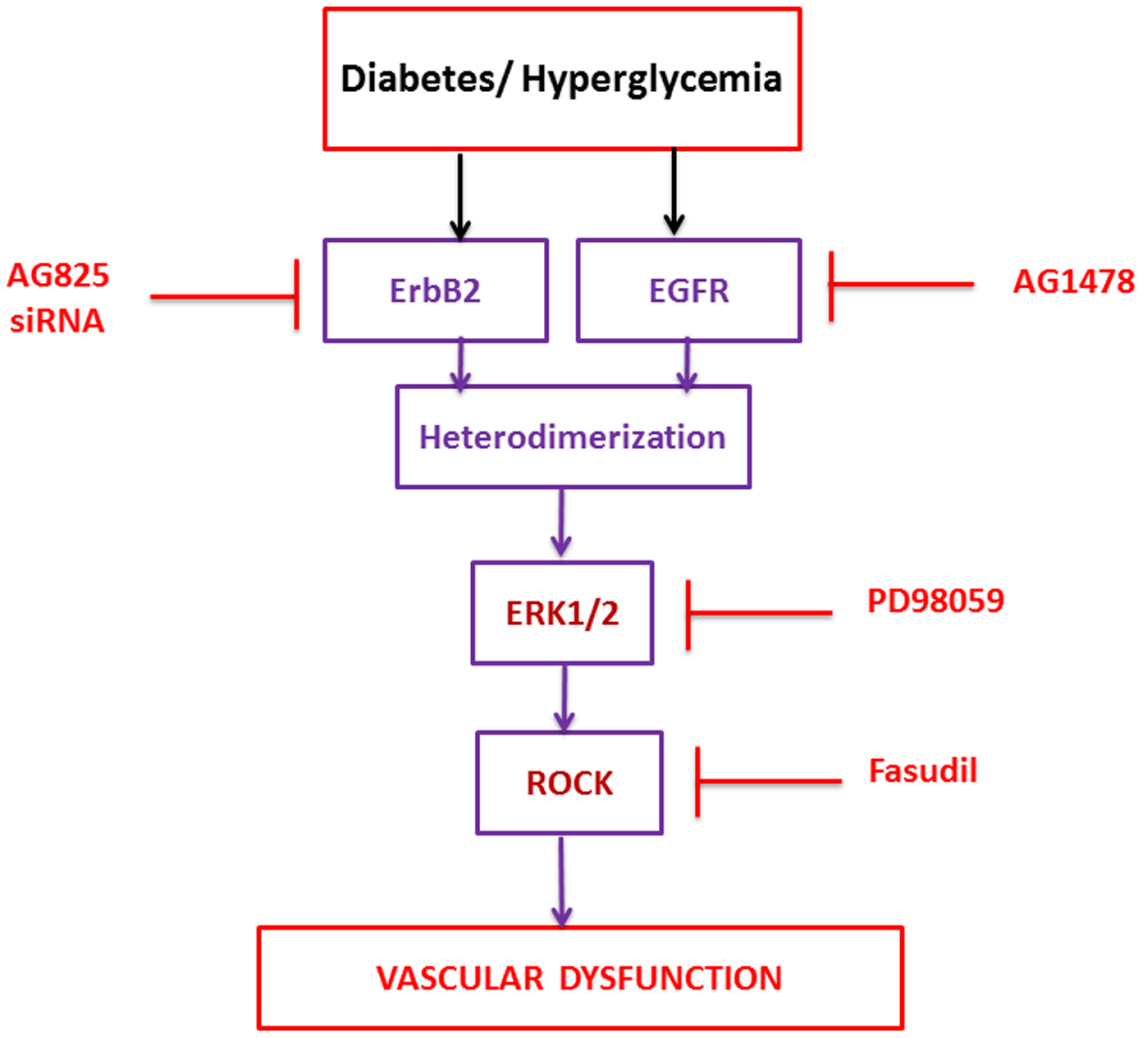
Schematic model of how ErbB2 via heterodimerization with EGFR and downstream signaling via ERK1/2 and ROCKs might mediate diabetes-induced dysfunction. The pharmacological inhibitors used in this study are also shown (see text for further explanation).

It was not clear from the literature whether ERK1/2 and ROCK signaling is actually coupled in the vasculature and if it is, which is the upstream effector. To address this, we examined the impact of ROCK II or ERK1/2 blockade on the phosphorylation of the two molecules following either acute treatment of the isolated diabetic mesenteric vascular bed or in cultured VSMC grown in high glucose. The fact that in both the intact vasculature and in cultured VSMC, PD98059 led to marked inhibition of ROCK II and ERK1/2 phosphorylation whereas fasudil only led to inhibition of ROCK II phosphorylation (Figure 10), even with the caveat that fasudil might also have off-target effects on other kinases such as protein kinase A, suggests that ERK1/2 are likely upstream effectors of ROCKs. Thus, collectively our data from chronically treated animals, acute treatments of the isolated mesenteric vascular bed, and from cultured VSMC grown in high glucose, provide strong evidence that activation of ErbB2, heterodimer formation with EGFR, and downstream signaling via ERK1/2-ROCKs as an important pathway mediating vascular dysfunction associated with diabetes (see our working model in Figure 11).

As to what might be the precise upstream inputs and downstream effectors of this novel ErbB2/EGFR-ERK1/2-ROCK pathway in mediating diabetes-induced vascular dysfunction are not clear. Although multifactorial and complex mechanisms are likely involved [4]–[5] potential upstream effectors might include G-protein coupled receptors of the renin-angiotensin-aldosterone system (RAAS). Ang II, and Aldosterone levels appear raised in diabetes [33]–[34] and are also known transactivators of EGFR [11], [13]–[14], [35], and possibly ErbB2 [13], via src and MMP-dependent mechanisms [14]. Consistent with these findings, we have recently shown that glucose-mediated transactivation occurs via a src-dependent mechanism and its blockade by several strategies prevents diabetes-induced vascular dysfunction [11].

Oxidative- and endoplasmic reticulum (ER)-stress are also potential upstream effectors of the ErbB family of receptors [5], [36]–[37]. Oxidative stress such as that induced by H_2_O_2_ is known to transactivate EGFR [38]. Ang II is also thought to induce oxidative stress via activation of NADPH oxidase [39]–[41] that eventually leads to ER-stress [42] and might represent an alternative mechanism by which Ang II might switch-on ErbB receptor signaling. Indeed, recent report suggests that EGFR-induced ER-stress might lead to vasculature dysfunction and cardiac fibrosis in experimental diabetes [20].

The possibility that Ang II might be an upstream effector of ErbB2 signaling is supported by the fact that Ang II, rather like erbB2 in this study, can also upregulate ERKs and ROCK - a regulator of diverse vascular functions including smooth muscle contraction, cell migration and adhesion, and cell inflammatory responses all of which may be involved in vascular dysfunction such as atherosclerosis [23], [43]–[44]. Elevated ROCK activity may lead to vascular dysfunction through several downstream mechanisms including modulation of contractile machinery and regulation of eNOS [23]–[24] -though these were not studied here. It is clear that inhibition of ROCK, as in the present study, as well as by other groups [25]–[26], [31] confirms the importance of Rho/ROCK signaling in vascular function and its inhibitors may represent interesting therapies for diabetes-induced vascular complications. Indeed, the clinical benefits of “statins” (HMG-CoA reductase inhibitors) in diabetic cardiovascular complications are thought to occur partly via inhibition of ROCKs as consequence of their ability to not only inhibit cholesterol biosynthesis but also decrease the formation of isoprenoid intermediates required for the activation of ROCKs [23], [45]. Since “statins” such as Lovastatin are also known to inhibit EGFR signaling [27], our study therefore suggests that statins might also, at least in part, function by inhibiting ROCKs via blockade of the ErbB2/EGFR-ERK1/2 pathway- a possibility that requires further study. Thus, targeting of this novel ErbB2/EGFR-ERK1/2/ROCK pathway in diabetes-induced vascular complications might be clinically useful especially when there is already a vast experience with the use of ErbB2 inhibitors in several cancers [10], [30]. However, this should be cautioned with the knowledge that ErbB2, rather like EGFR, may have differing roles in other tissues [6]. For example, we have recently shown that signaling via ErbB2, in contrast to its detrimental role in vascular dysfunction described in the present study, may actually have a beneficial role in facilitating cardiac recovery from ischemia-reperfusion injury in diabetes [28]. Indeed, ErbB2 blockade with certain monoclonal antibody inhibitors can lead to cardiac toxicities in cancer patients [46]–[48] and thus, use of delivery systems or other targeting strategies that allow selective blockade of erbB2 in the vasculature may be required for clinical applications.

In addition to the potential upstream of role of Ang II and other RAAS members, we speculate that diabetes and/or hyperglycemia might activate ErbB2 signaling via other, as yet, undefined pathways as well. This is supported by recent clinical trials studies that have suggested that Ang II (Type 1A) receptor blockade (e.g. with Losartan) does not completely reverse vascular dysfunction [49]. Further, simple correction of hyperglycemia *per se* also does not completely prevent development of vascular complications in patients with diabetes [50] implying that other factors associated with the diabetic state could induce ErbB2/EGFR/ERK1/2/ROCK signaling- a possibility that needs further study.

In conclusion, this study has identified a novel signaling mechanism leading to diabetes-induced vascular dysfunction that involves activation of ErbB2 receptor tyrosine kinase, heterodimer formation with EGFR and downstream signaling via ERK1/2 and ROCK (see Figure 11). Thus, our data suggests that potential strategies aimed at modifying actions of signal transduction pathways involving ErbB2 pathway may prove to be beneficial in the treatment of diabetes-induced vascular complications.
